# Formononetin Alleviates Cigarette Smoke Extract-Induced Lung Injury in Mice with Gut Microbiota and Taurine/SLC6A6/NF-κB Modulation

**DOI:** 10.3390/molecules31173090

**Published:** 2026-09-03

**Authors:** Zheng Ma, Sen Hu, Zhongting Lv, Shuang Liu, Jia Yu, Jie Zhang, Hui Tian, Li Ren

**Affiliations:** 1 Department of Thoracic Surgery, Qilu Hospital of Shandong University, Jinan 250012, China; 2 College of Food Science and Engineering, Jilin University, Changchun 130062, China

**Keywords:** formononetin, cigarette smoke extract, gut–lung axis, taurine metabolism

## Abstract

Cigarette smoke exposure is a major preventable risk factor for respiratory disease and drives oxidative and inflammatory lung injury, highlighting the urgent need for safe, multi-target interventions beyond conventional anti-inflammatory therapy. Formononetin (FMN), a naturally occurring isoflavone with antioxidant, anti-inflammatory, and microbiota-modulating potential, may offer a dietary-botanical strategy against smoke-related pulmonary damage. This study evaluated FMN against cigarette smoke extract (CSE)-induced lung injury and explored its associations with intestinal barrier integrity, gut microbiota, taurine metabolism, SLC6A6 expression, and NF-κB activation. In a CSE-challenged mouse model, oral FMN, particularly at 70 mg/kg, improved body weight gain, reduced the lung index, attenuated pulmonary histopathological injury, restored GSH/GSSG and SOD, decreased MDA, and suppressed TNF-α, IL-1β, and IL-6 expression. FMN also ameliorated colonic injury and restored ZO-1 and occludin expression. Exploratory 16S rRNA sequencing and fecal metabolomics indicated FMN-associated alterations in gut microbial composition and metabolic profiles, with taurine and hypotaurine metabolism identified as a candidate pathway for further investigation. FMN further restored lung taurine content and SLC6A6 expression while inhibiting NF-κB activation. These findings suggest that FMN alleviates CSE-induced lung injury and that this improvement is associated with coordinated changes in gut microbiota composition, taurine metabolism, SLC6A6 expression, and NF-κB activation.

## 1. Introduction

Cigarette smoke is a complex noxious aerosol rather than a single toxicant, and it continues to be one of the most preventable drivers of chronic respiratory morbidity and mortality worldwide [1,2]. WHO estimates indicate that tobacco causes more than 7 million deaths each year, including nearly 1.6 million deaths among non-smokers exposed involuntarily to second-hand smoke [1]. Although smoking cessation and anti-inflammatory treatment remain central to clinical management, few available interventions can concurrently blunt redox imbalance [2], inflammatory escalation [3], and smoke-induced disruption of mucosal homeostasis [4]. This has prompted growing interest in dietary and plant-derived bioactive compounds that may attenuate smoking-associated lung injury while reshaping related metabolic networks.

Oxidative imbalance and inflammatory activation are closely involved in lung injury induced by cigarette smoke extract (CSE) [2,3,4,5]. Oxidants and electrophilic chemicals derived from cigarette smoke may weaken endogenous antioxidant systems, accelerate lipid peroxidation, and trigger transcriptional programs sensitive to redox disturbance [2,3,5,6]. Among these pathways, nuclear factor-κB (NF-κB) acts as a key inflammatory hub by driving the production of mediators such as tumor necrosis factor-α (TNF-α), interleukin-1β (IL-1β), and interleukin-6 (IL-6) [5,6,7]. However, smoke-evoked injury should not be regarded as a purely pulmonary event. The gut–lung axis offers a mechanistic link between intestinal barrier function, microbial ecology, microbial-derived molecules, host metabolic outputs, and immune responses in the airway [8,9]. Taurine is of particular relevance in this setting, as it contributes to antioxidant protection [10], osmotic balance, mitochondrial maintenance [11], and the resolution of inflammation [12]. Previous studies have shown that taurine supplementation can alleviate oxidative stress and inflammatory responses. Cellular taurine availability is mainly controlled by the taurine transporter SLC6A6/TauT, a sodium- and chloride-dependent transporter responsible for taurine uptake and stress adaptation [13]. Accordingly, disturbance of the gut microbiota–taurine metabolism–SLC6A6/NF-κB regulatory axis may provide a less explored explanation for how intestinal dysbiosis and metabolic disorder contribute to smoke-associated pulmonary inflammation [6,8,9].

Food-derived flavonoids and botanical isoflavones are increasingly recognized as pleiotropic regulators of oxidative injury, inflammatory signaling, gut microbial homeostasis, and host metabolism [14,15,16]. Formononetin (FMN), or 7-hydroxy-4′-methoxyisoflavone, is a naturally occurring O-methylated isoflavone present in leguminous plants and in several medicinal or edible botanicals, including red clover and Astragalus species [17,18]. Structurally, the FMN aglycone contains no stereogenic center and therefore does not occur as R/S stereoisomers; isoformononetin, in which the hydroxy and methoxy substituents are interchanged, is a positional isomer rather than a stereoisomer. Existing experimental evidence suggests that FMN exerts antioxidant and anti-inflammatory effects in a range of disease-related models [17,18]. In pulmonary models, FMN mitigates cigarette smoke-evoked COPD-like damage by restraining inflammatory responses, endoplasmic reticulum stress, and epithelial cell apoptosis, partly via AhR/CYP1A1- and AKT/mTOR-associated signaling [19]. It has also been shown to reduce airway inflammation and redox imbalance in mouse models of allergic asthma [20]. Collectively, these data support FMN as a potential phytochemical candidate against smoke-associated pulmonary injury [19,20]. However, most existing studies have focused primarily on lung-intrinsic signaling events, while the contribution of intestinal barrier function, gut microbial remodeling, and taurine-associated metabolic regulation remains insufficiently characterized.

Accordingly, this work examined the protective effect of FMN against CSE-evoked lung injury and explored its potential link to modulation of the gut microbiota–taurine metabolism–SLC6A6/NF-κB axis. To this end, a CSE-induced mouse model was used to evaluate pulmonary histopathology, oxidative stress, inflammatory cytokine expression, intestinal barrier integrity, gut microbiota composition, metabolomic alterations, lung taurine content, SLC6A6 expression, and NF-κB activation. We hypothesized that FMN would attenuate CSE-induced pulmonary oxidative and inflammatory injury in association with preservation of intestinal barrier integrity, correction of gut microbial and metabolic disturbances, restoration of taurine/SLC6A6 homeostasis, and suppression of NF-κB-mediated inflammatory activation.

## 2. Results

### 2.1. FMN Mitigated CSE-Evoked Pulmonary Damage, Oxidative Stress, and Inflammatory Responses in Mice

**Figure 1:**
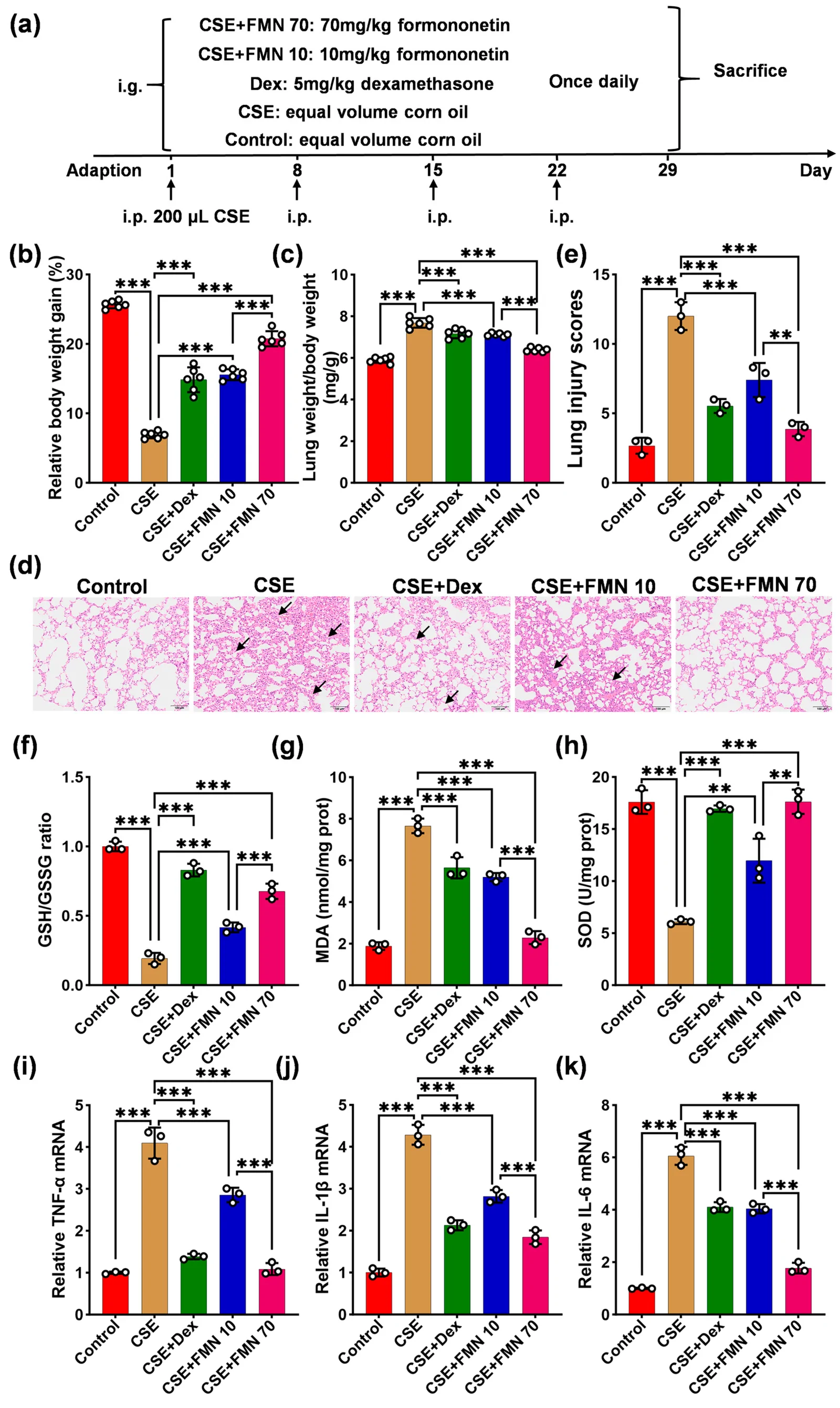
Formononetin attenuates CSE-induced lung injury, oxidative stress, and inflammatory responses in mice. (**a**) Schematic illustration of experimental design. (**b**) Relative body weight gain in each group (n = 6). (**c**) Lung weight/body weight ratio (n = 6). (**d**) Representative histopathological images of lung tissues (n = 3). Black arrows indicate representative areas of inflammatory-cell infiltration and alveolar septal thickening. Scale bars = 100 μm. (**e**) Lung injury scores (n = 3). (**f**–**h**) Oxidative stress-related parameters in lung tissues, including the GSH/GSSG ratio, MDA level, and SOD activity (n = 3). (**i**–**k**) Relative mRNA expression levels of the pro-inflammatory cytokines TNF-α, IL-1β, and IL-6 in lung tissues (n = 3). CSE, cigarette smoke extract; FMN, formononetin; Dex, dexamethasone. Data are presented as mean ± SD. Statistical significance is indicated by brackets: ** *p* < 0.01, *** *p* < 0.001. Statistics: one-way ANOVA followed by Tukey’s test for panels (**b**,**c**,**e**–**k**).

To assess FMN-mediated protection against CSE-evoked pulmonary injury, mice received intraperitoneal CSE injections on days 1, 8, 15, and 22, together with daily oral FMN at 10 or 70 mg/kg [21]. The doses were selected based on previous in vivo pharmacological studies of FMN, with 10 mg/kg representing a low dose and 70 mg/kg representing a higher dose within the previously reported effective range of 10–100 mg/kg. Dexamethasone (Dex, 5 mg/kg) served as the positive control [22], and all animals were euthanized on day 29 for further analyses (Figure 1a).

Compared with the Control group, mice exposed to CSE showed a clear reduction in relative body weight gain, accompanied by an increased lung weight-to-body weight ratio. These changes are consistent with impaired growth and CSE-evoked pulmonary swelling or inflammatory enlargement. Administration of Dex or FMN partly reversed these alterations, as reflected by improved weight gain and a lower lung index. The protective effect was more evident in the FMN 70 mg/kg group than in the low-dose FMN group (Figure 1b,c).

Histological assessment yielded a similar pattern. Lung sections from Control mice showed well-preserved alveolar architecture, thin interalveolar septa, and open airspaces. In contrast, CSE exposure caused overt structural injury, including alveolar disruption, inflammatory cell infiltration, and septal thickening. Both Dex and FMN attenuated these pathological changes, with the most apparent improvement observed in the CSE + FMN 70 group. Consistently, CSE exposure increased the lung injury score, whereas Dex and FMN treatment reduced the histopathological index (Figure 1d,e).

We then assessed whether FMN mitigated oxidative damage in lung tissue after CSE exposure. CSE decreased the GSH/GSSG ratio and SOD activity, while increasing MDA content, indicating disturbed redox homeostasis and enhanced lipid peroxidation. Dex and FMN treatment restored antioxidant capacity, as shown by increased GSH/GSSG ratio and SOD activity together with reduced MDA accumulation. Among the FMN-treated groups, 70 mg/kg produced a stronger antioxidant response than 10 mg/kg, with a higher GSH/GSSG ratio, lower MDA level, and greater SOD activity (Figure 1f–h).

CSE challenge also markedly increased pulmonary mRNA abundance of TNF-α, IL-1β, and IL-6 relative to the Control group. This cytokine induction was blunted by Dex and FMN, with 70 mg/kg FMN producing greater repression than 10 mg/kg FMN (Figure 1i–k). Collectively, these data indicate that FMN mitigates CSE-evoked lung damage, partly through dampening oxidative stress and inflammatory activation.

### 2.2. FMN Ameliorated CSE-Induced Colonic Injury and Restored Intestinal Barrier Integrity

**Figure 2:**
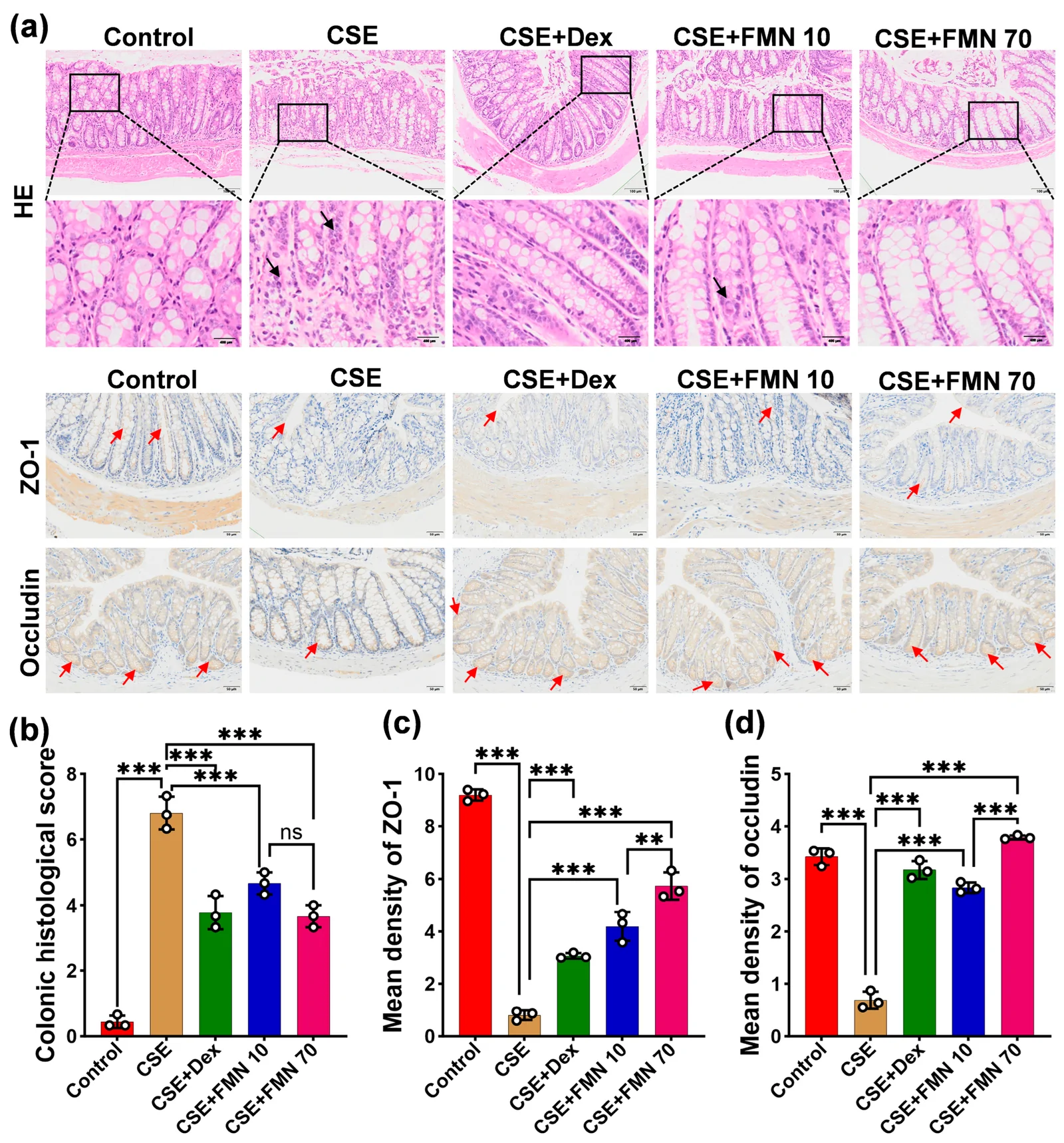
FMN ameliorates CSE-induced colonic injury and restores intestinal barrier-related tight junction protein expression. (**a**) Representative H&E-stained and immunohistochemically stained colon sections from each group. In the enlarged H&E images, black arrows indicate representative crypt distortion and inflammatory-cell infiltration. In the immunohistochemical images, red arrows indicate representative brown DAB-positive epithelial staining for ZO-1 or occludin. Scale bars: 100 μm (low-magnification H&E), 40 μm (enlarged H&E), and 50 μm (immunohistochemistry). (**b**) Colonic histological scores. (**c**,**d**) Quantitative analysis of the mean density of ZO-1 and occludin immunostaining. Data are presented as mean ± SD, with individual dots representing independent samples; n = 3 per group. Statistical significance is indicated by brackets: ** *p* < 0.01, *** *p* < 0.001; ns, not significant. Statistics: one-way ANOVA followed by Tukey’s test for panels (**b**–**d**).

Given the potential involvement of the gut–lung axis in CSE-induced lung injury, we next examined whether FMN could protect against CSE-induced colonic damage and intestinal barrier disruption. Low- and high-magnification H&E images showed that the Control group had a continuous surface epithelium, regularly aligned and closely packed crypts, numerous goblet-cell profiles, and minimal inflammatory-cell infiltration. In contrast, CSE exposure produced marked mucosal injury characterized by epithelial disruption, shortened and disorganized crypts, fewer visible goblet-cell profiles, and prominent inflammatory-cell infiltration in the lamina propria. Dex and FMN treatment preserved epithelial continuity and crypt architecture and reduced inflammatory infiltration, with the CSE + FMN 70 group showing the most evident structural recovery among the intervention groups (Figure 2a). Consistent with these morphological observations, the colonic histological score was markedly increased by CSE and significantly reduced by Dex and both FMN doses (Figure 2b).

We next evaluated intestinal barrier integrity by immunohistochemical staining for the tight-junction proteins ZO-1 and occludin. Control colon tissues displayed comparatively high epithelial immunoreactivity for both proteins, whereas CSE exposure caused a pronounced reduction in the staining signals. Dex and FMN restored ZO-1 and occludin immunoreactivity to different extents. Quantitative analysis confirmed that the mean densities of ZO-1 and occludin were significantly decreased in the CSE group and increased after Dex or FMN treatment, with the strongest restoration observed in the CSE + FMN 70 group (Figure 2c,d). Together, these morphological and quantitative findings indicate that FMN alleviates CSE-induced colonic injury and supports intestinal barrier integrity.

### 2.3. Formononetin Reshaped the Gut Microbiota Composition in Mice with CSE-Induced Lung Injury

**Figure 3:**
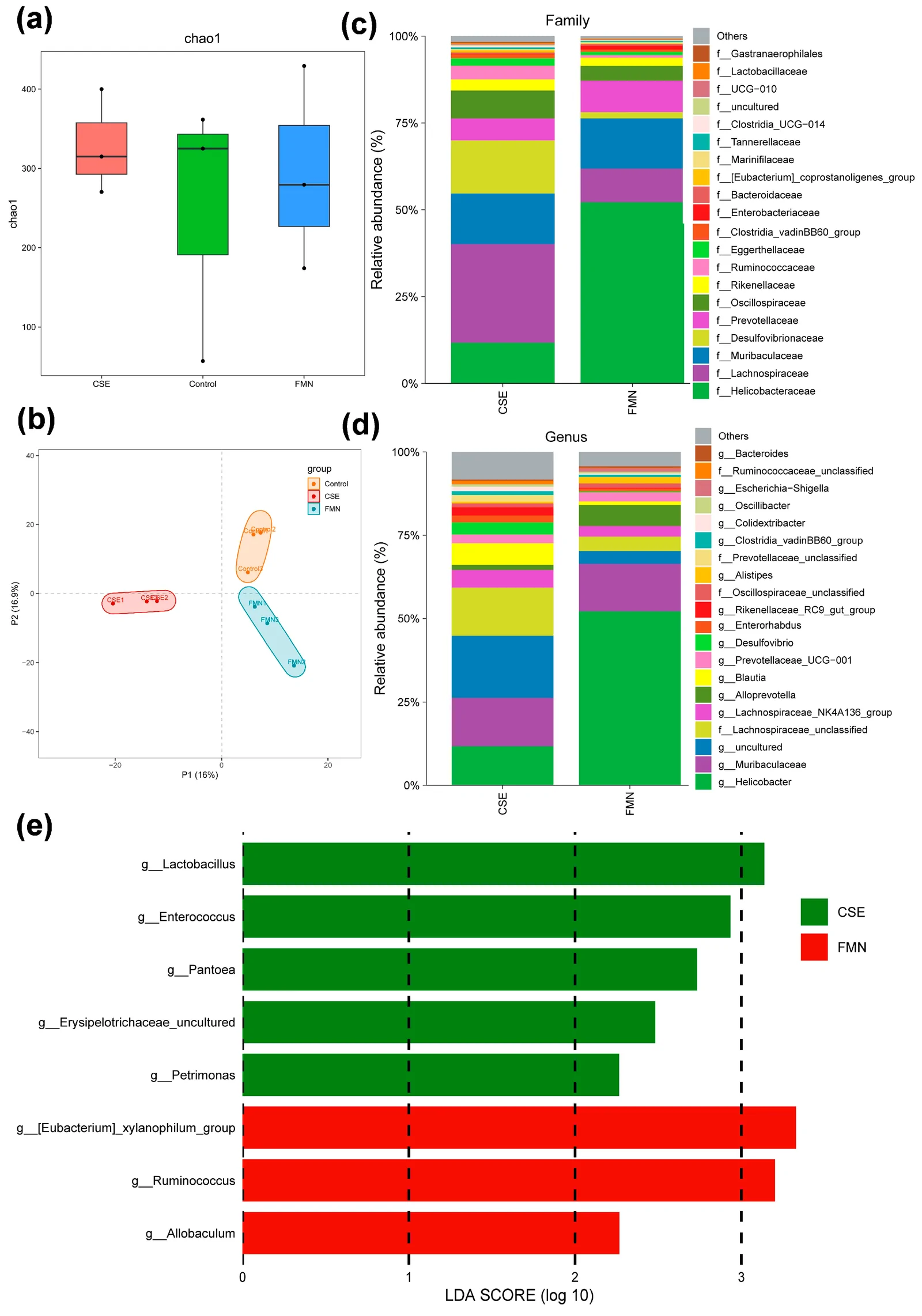
Formononetin modulates the gut microbiota composition in mice with CSE-induced lung injury. Gut microbiota analysis was performed using n = 3 biologically independent samples per group from the Control, CSE, and CSE + FMN 70 groups. (**a**) Alpha diversity of the gut microbiota was evaluated using the Chao1 index. (**b**) PCoA analysis showing differences in beta diversity among the Control, CSE, and 70 mg/kg FMN intervention groups. (**c**,**d**) Relative abundance of gut microbiota at the family and genus levels in the CSE and FMN groups. (**e**) LEfSe analysis showing differentially enriched bacterial genera between the CSE and FMN groups, presented as LDA scores. Data are presented as mean ± SD, with individual dots representing independent samples. Statistics: one-way ANOVA/Tukey’s test for panel (**a**); panels (**b**–**d**) are descriptive; LEfSe in panel (**e**) used the Kruskal–Wallis test and LDA (*p* < 0.05, LDA score > 2.0).

Since FMN improved CSE-induced colonic injury and intestinal barrier disruption, we further investigated whether its protective effect was associated with alterations in gut microbiota composition. Alpha diversity was first evaluated using the Chao1 index. As shown in Figure 3a, no obvious difference in microbial richness was observed among the Control, CSE, and FMN groups, suggesting that CSE exposure and FMN intervention did not markedly alter the overall richness of gut microbiota.

We next performed principal coordinate analysis (PCoA) to assess differences in microbial community structure. The PCoA plot showed a clear separation among the Control, CSE, and FMN groups, indicating that CSE exposure induced a distinct shift in gut microbiota composition. Notably, samples from the FMN group were clearly separated from those in the CSE group, suggesting that FMN intervention reshaped the CSE-disrupted gut microbial profile (Figure 3b).

To further characterize the microbial alterations induced by FMN, we analyzed the relative abundance of gut microbiota at the family and genus levels. At the family level, the microbial composition differed markedly between the CSE and FMN groups, with apparent changes in the relative abundance of dominant bacterial families, including *Helicobacteraceae*, *Lachnospiraceae*, *Muribaculaceae*, *Prevotellaceae*, *Desulfovibrionaceae*, and *Rikenellaceae* (Figure 3c). At the genus level, FMN treatment also altered the relative abundance distribution of several major bacterial genera, including *Helicobacter*, *Muribaculaceae_unclassified*, *Lachnospiraceae_unclassified*, *Alloprevotella*, *Blautia*, and *Desulfovibrio* (Figure 3d).

Linear discriminant analysis effect size (LEfSe) analysis was then performed to identify candidate bacterial taxa contributing to the discrimination between the CSE and FMN groups. The results showed that *Lactobacillus*, *Enterococcus*, *Pantoea*, *Erysipelotrichaceae_uncultured*, and *Petrimonas* were enriched in the CSE group, whereas *[Eubacterium]_xylanophilum_group*, *Ruminococcus*, and *Allobaculum* were enriched in the FMN group (Figure 3e). Collectively, these findings indicate that FMN does not substantially alter microbial richness but markedly reshapes the overall gut microbiota structure and specific bacterial taxa in mice with CSE-induced lung injury.

### 2.4. Exploratory Metabolomic Profiling Identified FMN-Associated Metabolic Changes in CSE-Treated Mice

**Figure 4:**
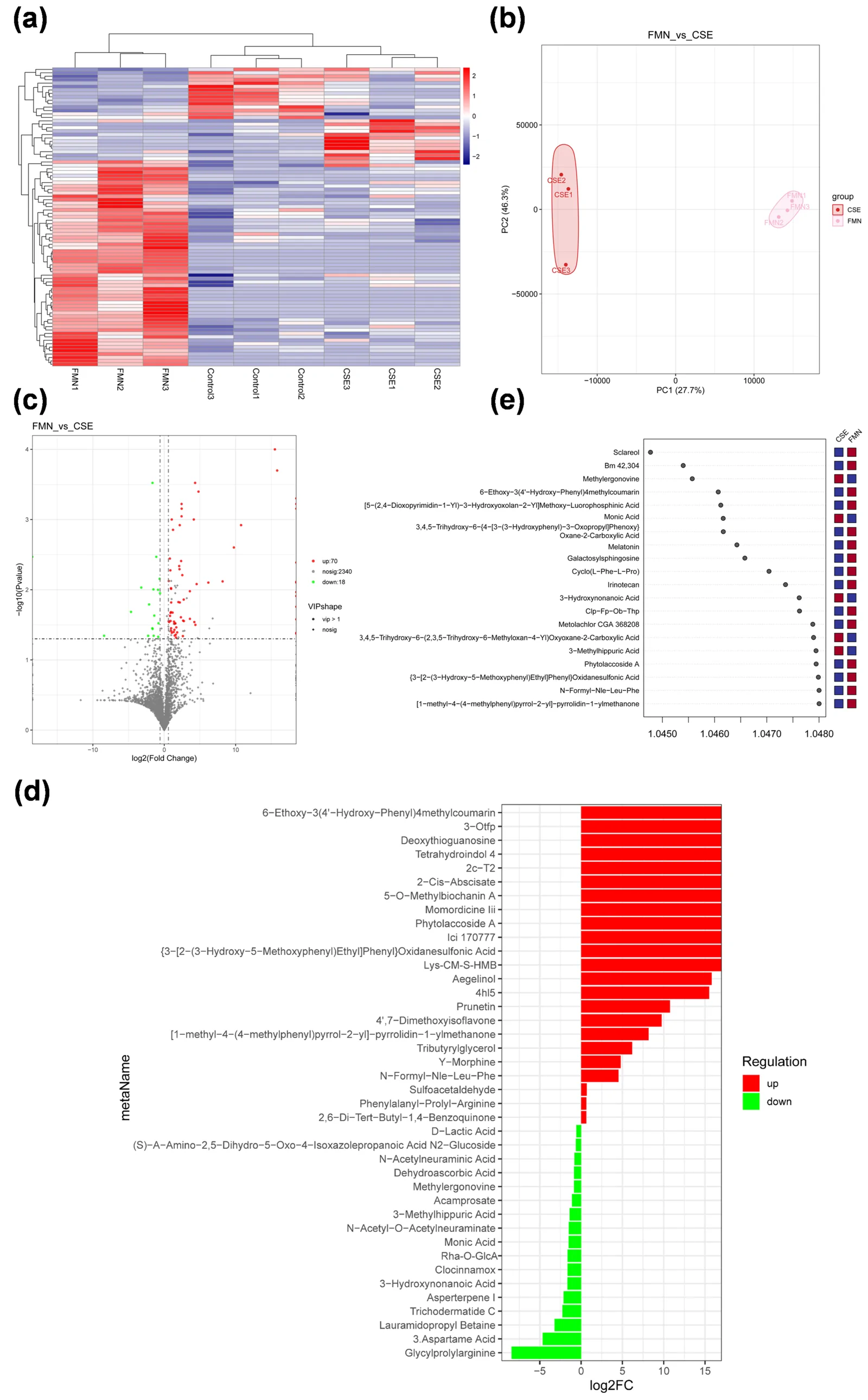
Formononetin modulates metabolic alterations in mice with CSE-induced lung injury. Metabolomic analysis was performed using n = 3 biologically independent samples per group from the Control, CSE, and CSE + FMN 70 groups. (**a**) Hierarchical clustering heatmap of differential metabolites among the Control, CSE, and FMN groups. Red indicates increased relative abundance, whereas blue indicates decreased relative abundance. (**b**) PCA score plot showing a clear separation of metabolic profiles between the CSE and FMN groups. (**c**) Volcano plot of differential metabolites between the FMN and CSE groups. Red and green dots indicate significantly upregulated and downregulated metabolites, respectively, while gray dots indicate metabolites with no significant difference. (**d**) Bar plot showing representative differential metabolites ranked by log2FC in the FMN group compared with the CSE group. (**e**) Representative metabolites ranked according to VIP scores. Data are presented as mean ± SD; n = 3 per group. Statistics: panels (**a**,**b**) are descriptive; differential metabolites in panels (**c**,**d**) were screened using |log2FC| > 1 and VIP > 1. Raw *p*-values were adjusted using the Benjamini–Hochberg procedure, and q < 0.05 was considered statistically significant. Features meeting only the nominal *p*-value threshold were regarded as exploratory candidates.

Given that FMN markedly reshaped the gut microbiota composition in CSE-treated mice, we next performed metabolomic analysis to determine whether FMN intervention was accompanied by metabolic reprogramming. Hierarchical clustering analysis revealed distinct metabolic patterns among the Control, CSE, and FMN groups. The relative abundance of multiple metabolites was markedly altered after CSE exposure, whereas FMN treatment induced a different metabolic profile, indicating that FMN could modulate CSE-associated metabolic disturbances (Figure 4a).

Principal component analysis (PCA) was further performed to evaluate the overall metabolic differences between the CSE and FMN groups. The score plot showed a clear separation between CSE-treated and FMN-treated samples along the principal components, suggesting that FMN intervention substantially changed the global metabolic phenotype of CSE-treated mice (Figure 4b). Consistently, volcano plot analysis identified a series of significantly altered metabolites between the FMN and CSE groups, including both upregulated and downregulated metabolites in response to FMN treatment (Figure 4c).

To further characterize these metabolic alterations, representative differential metabolites were ranked according to log2FC. Compared with the CSE group, FMN treatment increased the abundance of several metabolites, including 6-ethoxy-3-(4′-hydroxy-phenyl)-4-methylcoumarin, deoxythuganosine, tetrahydroindolizine 4, monordicine II, phytolaccoside A, and ageilhol, whereas metabolites such as glycyprolylarginine, aspartame I, lauramidopropyl betaine, trichodermatide C, and aspertene I were decreased after FMN intervention (Figure 4d). In addition, VIP score analysis further identified key metabolites contributing to the discrimination between the CSE and FMN groups, including sclareol, methylergonovine, galactosylsphingosine, cyclo-L-Phe-L-Pro, irinotecan, 3-hydroxynonanoic acid, and metolachlor CGA 368208 (Figure 4e).

Collectively, these exploratory findings indicate that FMN treatment was associated with changes in the fecal metabolic profile of CSE-treated mice. These metabolic changes provide an important basis for subsequent pathway enrichment analysis and suggest that specific metabolite-related pathways may participate in the protective effects of FMN against CSE-induced lung injury.

### 2.5. Taurine and Hypotaurine Metabolism Emerged as a Candidate Pathway Associated with FMN Treatment

**Figure 5:**
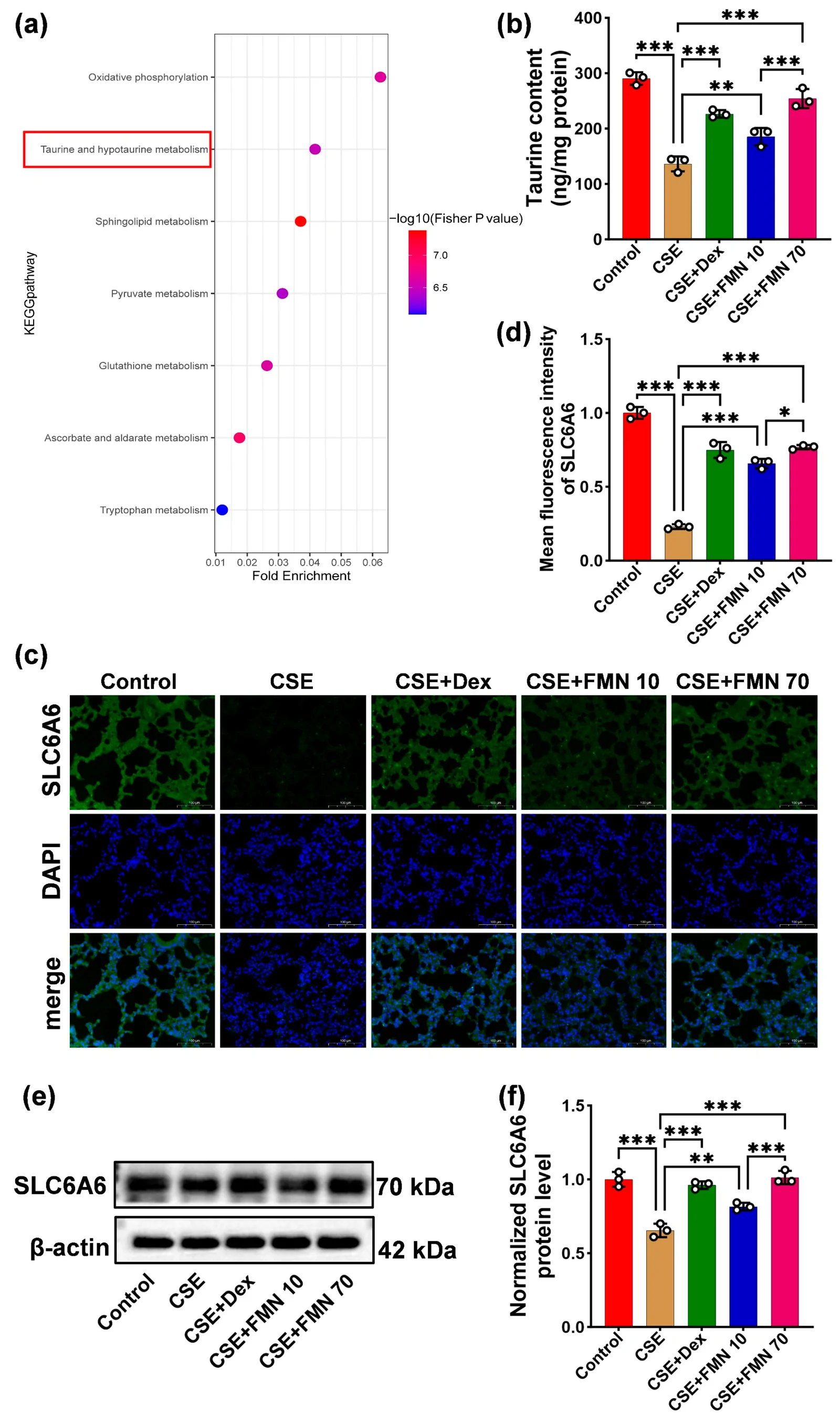
Formononetin regulates taurine metabolism and SLC6A6 expression in mice with CSE-induced lung injury. (**a**) KEGG pathway enrichment analysis of differential metabolites between the CSE and FMN groups. KEGG pathway enrichment analysis was performed using the differential metabolites identified from metabolomic datasets comprising three biologically independent samples per group. The taurine and hypotaurine metabolism pathway was significantly enriched and is highlighted with a red box. (**b**) Quantitative analysis of taurine content in lung tissues from each group (n = 3). (**c**) Representative immunofluorescence images of SLC6A6 in lung tissues. SLC6A6 is shown in green, and nuclei are counterstained with DAPI in blue (n = 3). Scale bars = 100 μm. (**d**) Quantification of the mean fluorescence intensity of SLC6A6 (n = 3). (**e**) Representative Western blot images of SLC6A6 protein expression in lung tissues, with β-actin used as the loading control (n = 3). (**f**) Quantitative analysis of normalized SLC6A6 protein levels (n = 3). Data are presented as mean ± SD; n = 3 per group. * *p* < 0.05, ** *p* < 0.01, *** *p* < 0.001. Statistics: Fisher’s exact test for panel (**a**); one-way ANOVA followed by Tukey’s test for panels (**b**,**d**,**f**).

To further identify the metabolic pathways involved in the protective effects of FMN against CSE-induced lung injury, KEGG pathway enrichment analysis was performed based on the differential metabolites between the CSE and FMN groups. The results showed that several metabolic pathways were significantly enriched, including oxidative phosphorylation, taurine and hypotaurine metabolism, sphingolipid metabolism, pyruvate metabolism, glutathione metabolism, ascorbate and aldarate metabolism, and tryptophan metabolism. Among these pathways, taurine and hypotaurine metabolism was selected for further validation because of its close association with oxidative stress regulation and inflammatory responses (Figure 5a).

We next measured taurine content in lung tissues. Compared with the Control group, CSE exposure significantly decreased taurine levels, suggesting that CSE-induced lung injury was accompanied by disruption of taurine metabolism. Treatment with Dex or FMN markedly restored taurine content in lung tissues. Notably, FMN at 70 mg/kg showed a stronger effect than FMN at 10 mg/kg, indicating a dose-related regulatory effect of FMN on taurine metabolism (Figure 5b).

Since SLC6A6 is an important taurine transporter involved in taurine homeostasis, we further examined SLC6A6 expression in lung tissues. Immunofluorescence staining showed strong SLC6A6 signals in the Control group, whereas CSE exposure markedly reduced SLC6A6 fluorescence intensity. Dex and FMN treatment significantly enhanced SLC6A6 immunofluorescence intensity compared with the CSE group, and the CSE + FMN 70 group exhibited a higher SLC6A6 signal than the CSE + FMN 10 group (Figure 5c,d).

Western blot analysis was then performed to further evaluate SLC6A6 protein expression. The results showed that CSE exposure altered SLC6A6 protein abundance in lung tissues, while Dex and FMN intervention significantly regulated SLC6A6 expression compared with the CSE group. No significant difference in normalized SLC6A6 protein levels was observed between the CSE + FMN 10 and CSE + FMN 70 groups (Figure 5e,f).

### 2.6. Formononetin Restored SLC6A6 Expression and Suppressed NF-κB-Mediated Inflammatory Activation

Having shown that FMN restored taurine levels and regulated the taurine transporter SLC6A6 in CSE-treated mice, we next examined whether this metabolic restoration was accompanied by suppression of inflammatory signaling. Since NF-κB is a central regulator of inflammatory cytokine production, the activation of the NF-κB pathway was evaluated in lung tissues.

**Figure 6:**
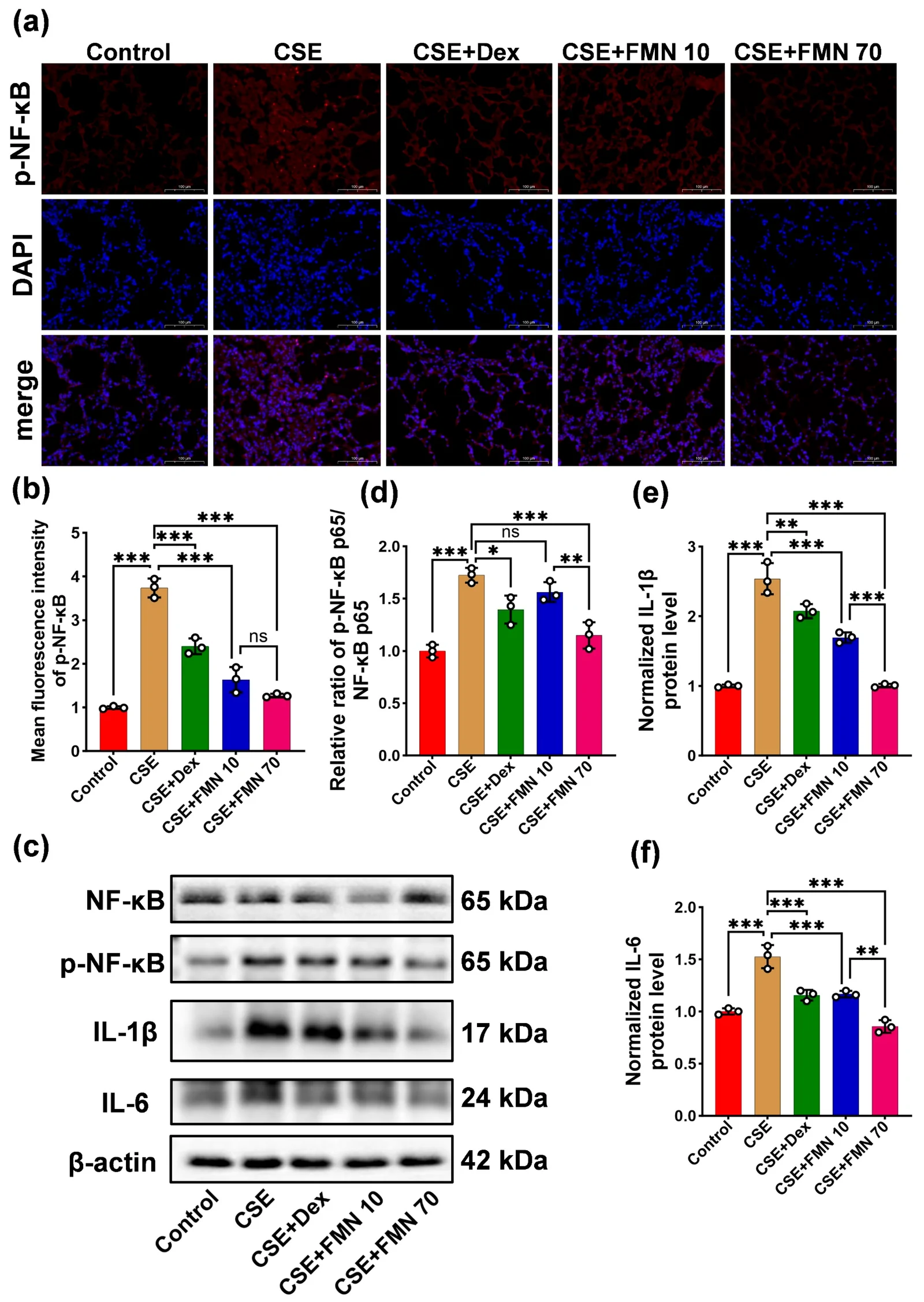
Formononetin suppresses activation of the NF-κB inflammatory signaling pathway in lung tissues of mice with CSE-induced lung injury. (**a**) Representative immunofluorescence images of p-NF-κB in lung tissues from each group. p-NF-κB is shown in red, and nuclei are counterstained with DAPI in blue (n = 3). Scale bars = 100 μm. (**b**) Quantification of the mean fluorescence intensity of p-NF-κB (n = 3). (**c**) Representative Western blot images of NF-κB, p-NF-κB, IL-1β, and IL-6 protein expression in lung tissues (n = 3). (**d**) Quantitative analysis of the p-NF-κB/NF-κB ratio (n = 3). (**e**,**f**) Quantitative analysis of normalized IL-1β and IL-6 protein levels (n = 3). Data are presented as mean ± SD; n = 3 per group. * *p* < 0.05, ** *p* < 0.01, *** *p* < 0.001; ns, not significant. Statistics: one-way ANOVA followed by Tukey’s test for panels (**b**,**d**–**f**).

Immunofluorescence staining showed that p-NF-κB fluorescence intensity was relatively low in the Control group, whereas CSE exposure markedly enhanced p-NF-κB signals in lung tissues. Treatment with Dex or FMN significantly reduced CSE-induced p-NF-κB fluorescence intensity, indicating that FMN inhibited NF-κB activation in the lungs. Although both FMN doses reduced p-NF-κB staining, no significant difference was observed between the CSE + FMN 10 and CSE + FMN 70 groups in the immunofluorescence quantification (Figure 6a,b).

Western blot analysis further confirmed the inhibitory effect of FMN on NF-κB pathway activation. Compared with the Control group, CSE exposure significantly increased the ratio of p-NF-κB p65 to total NF-κB p65, suggesting activation of NF-κB signaling. Dex and FMN treatment reduced the CSE-induced increase in the p-NF-κB/NF-κB ratio, with FMN at 70 mg/kg showing a more evident inhibitory effect than FMN at 10 mg/kg (Figure 6c,d).

We then assessed the downstream inflammatory cytokines IL-1β and IL-6 at the protein level. CSE exposure markedly increased IL-1β and IL-6 protein expression compared with the Control group, consistent with the activation of inflammatory responses. In contrast, Dex and FMN treatment significantly suppressed the expression of both IL-1β and IL-6. Notably, FMN at 70 mg/kg produced a stronger inhibitory effect than FMN at 10 mg/kg, especially for IL-1β and IL-6 protein levels (Figure 6e,f).

Together with the restoration of taurine metabolism and SLC6A6 expression shown above, these findings suggest that FMN-mediated protection against CSE-induced lung injury is associated with the regulation of the taurine/SLC6A6 axis and the suppression of NF-κB-mediated inflammatory activation.

### 2.7. Differential Gut Taxa Were Associated with Intestinal Barrier, Taurine/SLC6A6, and Inflammatory Indicators

To examine whether the FMN-associated microbial alterations were related to intestinal barrier integrity, redox homeostasis, taurine/SLC6A6 status, and pulmonary inflammatory activation, Spearman’s correlation analysis was performed using matched individual samples from the Control, CSE, and CSE + FMN 70 groups.

*[Eubacterium]_xylanophilum_group* was positively correlated with occludin mean density, the GSH/GSSG ratio, SOD activity, lung taurine content, and normalized SLC6A6 protein levels, while showing negative correlations with the p-NF-κB/NF-κB ratio and normalized IL-1β and IL-6 protein levels. *Ruminococcus* exhibited a similar correlation pattern, with positive associations with occludin, GSH/GSSG, SOD, lung taurine content, and SLC6A6, and a negative association with IL-1β. Allobaculum was positively correlated with occludin mean density.

**Figure 7:**
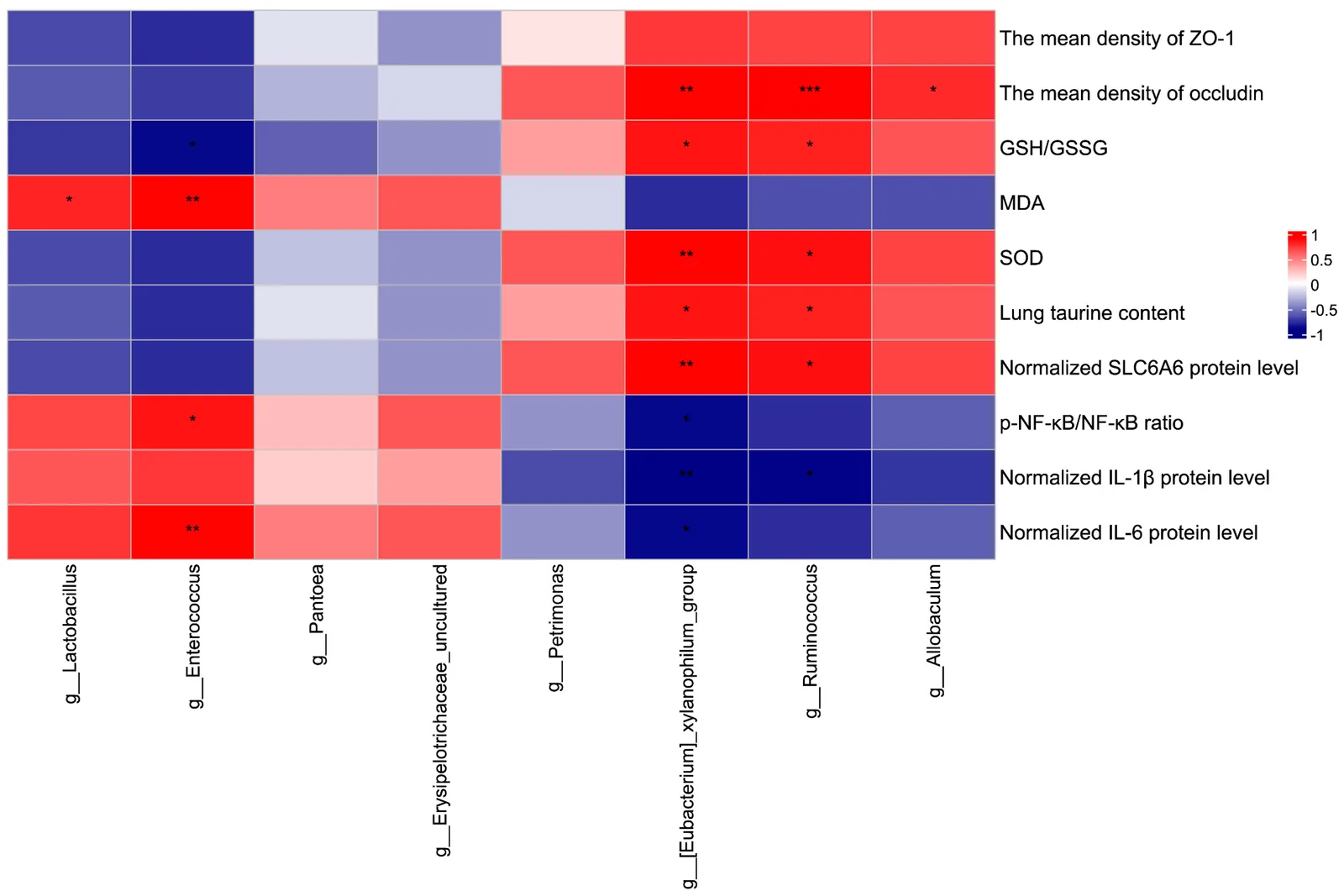
Correlations between selected differential gut bacterial genera and host physiological indicators. Correlations were calculated using n = 9 matched biological samples in total, comprising n = 3 samples per group from the Control, CSE, and CSE + FMN 70 groups. * *p* < 0.05, ** *p* < 0.01, *** *p* < 0.001.

In contrast, *Enterococcus* was negatively correlated with the GSH/GSSG ratio and positively correlated with MDA, the p-NF-κB/NF-κB ratio, and IL-6 protein expression. *Lactobacillus* was also positively correlated with MDA. These findings demonstrate coordinated associations between selected gut bacterial taxa and host barrier, oxidative, taurine/SLC6A6, and inflammatory phenotypes. However, these correlations do not establish direct or causal microbial–host relationships (Figure 7).

## 3. Discussion

The present study shows that formononetin (FMN), especially at 70 mg/kg, alleviates CSE-induced lung injury in mice. This protective effect was accompanied by improvement of pulmonary oxidative and inflammatory disturbances, preservation of intestinal barrier-related tight junction proteins, remodeling of gut microbiota composition, restoration of taurine-related metabolic alterations, regulation of SLC6A6, and suppression of NF-κB-associated inflammatory activation. These findings suggest that the pulmonary protective effect of FMN is associated with coordinated changes in the gut microbiota, taurine metabolism, SLC6A6 expression, and NF-κB activation.

Cigarette smoke-related lung injury is characterized by persistent oxidative pressure and inflammatory activation [2,3,4,5]. Cigarette smoke contains abundant oxidants and electrophilic compounds that disrupt epithelial redox buffering, promote lipid peroxidation, and activate pro-inflammatory transcriptional programs, among which NF-κB is a central regulatory node [2,5,6]. In line with this pathological framework, FMN improved both redox-associated and inflammation-associated abnormalities in CSE-exposed mice. Earlier studies have reported that FMN attenuates cigarette smoke-induced COPD-like lung injury by reducing inflammation, endoplasmic reticulum stress, and epithelial cell apoptosis, with involvement of AhR/CYP1A1 and AKT/mTOR signaling pathways [19]. FMN likewise mitigates airway inflammation and redox perturbation in allergic asthma models [20]. Unlike these earlier lung-centered studies, the present work simultaneously evaluated colonic barrier integrity, gut microbial composition, fecal metabolic profiles, pulmonary taurine content, SLC6A6 expression, and NF-κB activation. Thus, the novelty of this study lies not in re-demonstrating the established antioxidant and anti-inflammatory actions of FMN, but in placing these pulmonary effects within an underexplored gut microbiota–taurine metabolism–SLC6A6/NF-κB framework in CSE-induced injury.

The intestinal barrier findings provide an important extension of the lung-centered pharmacological interpretation. The lung and intestine are both mucosal organs exposed to environmental stimuli, and their communication through immune mediators, microbial components, and metabolites forms the biological basis of the gut–lung axis [8,9]. Cigarette smoke exposure has been associated with gut microbial dysbiosis, increased mucosal permeability, and altered intestinal immune responses [23,24]. In the present study, FMN preserved ZO-1 and occludin expression in colon tissues while improving smoke-related lung injury, suggesting that maintenance of intestinal epithelial integrity may be part of the systemic response associated with FMN intervention. This observation is consistent with the concept that intestinal barrier dysfunction can amplify extra-intestinal inflammatory responses and that barrier preservation may help restrain mucosal inflammatory crosstalk [8,9,23,24,25,26].

FMN also reshaped the gut microbial profile in CSE-treated mice. The separation of microbial communities after FMN intervention, together with the absence of a marked change in Chao1 richness, indicates that FMN mainly altered community structure rather than broadly increasing microbial richness. This pattern is consistent with the pharmacological behavior of many natural flavonoids, which tend to modulate specific microbial ecological states and metabolic outputs rather than uniformly increasing microbial diversity [14,15]. The enrichment of *Ruminococcus*, *Allobaculum*, and *[Eubacterium]_xylanophilum_group* after FMN treatment may reflect an ecological shift related to carbohydrate fermentation and mucosal metabolic homeostasis [27,28], although their functional contributions were not directly evaluated in the present study. Previous work has also shown that FMN can reshape the gut microbiota and improve host metabolic status in diet-induced metabolic disturbance models [29]. The microbial shifts observed here are correlated with systemic and pulmonary improvements, but their functional contribution requires dedicated validation.

The metabolomic data place the intestinal and pulmonary phenotypes within a broader pattern of host metabolic adjustment. After FMN treatment, the altered pathways included oxidative phosphorylation, glutathione metabolism, ascorbate and aldarate metabolism, sphingolipid metabolism, pyruvate metabolism, tryptophan metabolism, and taurine/hypotaurine metabolism. Functionally, these pathways point to changes in mitochondrial bioenergetics, redox buffering capacity, membrane lipid turnover, and immune–metabolic control. Taurine/hypotaurine metabolism is of particular interest in this setting, given the roles of taurine in antioxidant protection, osmotic balance, mitochondrial maintenance, epithelial stability, and inflammatory regulation. Recovery of taurine-related metabolism may therefore be one metabolic signature accompanying the protective effect of FMN against CSE-induced lung injury [10,11,12,13].

Taurine also offers a plausible biochemical link between metabolic reprogramming and inflammatory restraint. Under inflammatory conditions, taurine reacts with hypochlorous acid to generate taurine chloramine, a less injurious product with anti-inflammatory and cytoprotective activity. Through this reaction, taurine may help neutralize oxidant burden and prevent excessive activation of inflammatory signaling [10,11,30]. In our study, FMN restored pulmonary taurine levels while reducing NF-κB activation and the expression of downstream inflammatory cytokines. The concurrence of these changes suggests that recovery of taurine metabolism may help create a lung microenvironment less prone to persistent inflammatory escalation.

SLC6A6 provides a further point of mechanistic convergence between taurine availability and tissue taurine balance [13]. This sodium- and chloride-dependent transporter, also known as TauT, is required for cellular taurine uptake and for maintaining intracellular taurine pools during both basal and stress states. The FMN-associated regulation of SLC6A6 suggests that taurine transport, rather than taurine abundance alone, may participate in the adaptive metabolic response to CSE exposure. In this framework, SLC6A6 may act as an interface connecting taurine homeostasis with inflammatory signaling. The coordinated shifts in taurine content, SLC6A6 expression, and NF-κB activity are consistent with a potential link between regulated taurine transport and reduced smoke-induced pulmonary inflammation [31].

By integrating profiles of gut microbiota, fecal metabolism, and pulmonary molecular endpoints, our findings place the protective effects of FMN within a broader multi-level correlational framework, extending beyond its well-documented direct anti-inflammatory and antioxidant actions. Previous work has mainly characterized FMN as an anti-inflammatory, antioxidant, anti-apoptotic, or epithelial-protective agent in respiratory disease models. Here, we show that FMN-mediated lung protection occurs concomitantly with preservation of the intestinal barrier, remodeling of gut microbial ecology, restoration of taurine-related metabolism, and attenuation of NF-κB-dependent inflammatory activation. These correlated changes do not contradict earlier proposed mechanisms; rather, they describe a coordinated inter-organ pattern associated with FMN treatment in this model, without establishing a causal gut–metabolism–lung regulatory sequence.

This work has several caveats. First, the microbiome, metabolomics, taurine, SLC6A6, and NF-κB data are associative. The current design cannot establish the temporal sequence, necessity, or sufficiency of these events and therefore does not demonstrate that FMN acts through a gut microbiota–taurine metabolism–SLC6A6/NF-κB pathway. Functional studies using antibiotic-mediated microbiota depletion, fecal microbiota transplantation, taurine supplementation or depletion, and SLC6A6 inhibition, knockdown, or overexpression will be required to test the proposed relationship. Second, although two FMN doses were evaluated for the major pathological and biochemical endpoints, the microbiome and metabolomic analyses included only the Control, CSE, and CSE + FMN 70 groups. Because the 10 mg/kg FMN group was not included in the omics analyses, the observed microbial and metabolic changes cannot be interpreted as dose-dependent or generalized across FMN doses. These findings should therefore be regarded as exploratory observations associated with the 70 mg/kg treatment. Third, although the intraperitoneal CSE model reproduces selected oxidative, inflammatory, and histopathological features of smoke-related lung injury, it differs from chronic inhalation exposure and does not fully reproduce the progressive and heterogeneous characteristics of human COPD. Therefore, the present findings should be regarded as preclinical evidence, and further validation in chronic cigarette-smoke inhalation models is required before clinical implications can be considered. Finally, the microbiome and metabolomic analyses were based on only three independent biological samples per group. Therefore, individual bacterial taxa, metabolites, and enriched pathways should be interpreted as hypothesis-generating candidates rather than confirmed drivers of FMN-mediated protection. This limited sample size reduces statistical power, increases sensitivity to interindividual variation, and may increase the risks of both false-positive and false-negative findings. Although multiple-testing correction was applied, the omics results should be considered exploratory and require validation in larger independent cohorts.

## 4. Materials and Methods

### 4.1. Chemicals and Reagents

Formononetin (CAS No. 485-72-3) and dexamethasone, both with purity ≥ 98%, were obtained from Yuanye Bio-Technology Co., Ltd. (Shanghai, China). The chemical structure of formononetin is presented in Figure S1 of the Supplementary Materials. Assay kits for GSH/GSSG, MDA and SOD were supplied by Nanjing Jiancheng (Nanjing, China). The taurine ELISA kit was obtained from Mbio (Shanghai, China).

### 4.2. Preparation of CSE

CSE was generated using a modified published protocol [32,33]. Mainstream smoke generated from one commercial Huangguoshu cigarette was drawn through 2 mL of sterile PBS to obtain freshly prepared CSE solution. The extract was then titrated to pH 7.4, sterilized through a 0.22 μm membrane filter, and applied immediately.

### 4.3. Animals and Experimental Design

Male BALB/c mice aged 4–6 weeks and weighing 18–20 g were obtained from Changchun Yisi Laboratory Animal Co., Ltd. (Changchun, China). Animals were wild-type, non-genetically modified mice and had not undergone any previous experimental procedures. The animals were housed under specific pathogen-free conditions at 25 ± 1 °C with 60% relative humidity and a 12 h light/dark cycle, with free access to standard chow and water. All animal procedures were approved by the Institutional Animal Care and Use Committee of Jilin University under approval number 2025-317.

After acclimatization for 7 days, mice were randomly divided into five groups (n = 6 per group): Control, CSE, CSE + Dex, CSE + FMN 10, and CSE + FMN 70. Mice in the CSE, CSE + Dex, CSE + FMN 10, and CSE + FMN 70 groups were intraperitoneally injected with 200 μL CSE on days 1, 8, 15, and 22. Control mice received an equal volume of sterile PBS by intraperitoneal injection on the same days. During the intervention period, mice in the CSE + FMN 10 and CSE + FMN 70 groups were orally administered FMN at 10 and 70 mg/kg, respectively, once daily. Mice in the CSE + Dex group received Dex at 5 mg/kg once daily as a positive control. Control and CSE model mice received an equal volume of corn oil by oral gavage. All mice were sacrificed on day 29 for sample collection.

The individual mouse was defined as the experimental unit. A total of 30 mice were used and allocated as six mice per group. Sample size was based on previous studies using CSE-induced lung injury models and the expected magnitude of changes in lung injury and inflammatory endpoints. Animals were monitored daily for general appearance, activity, food and water intake, respiratory distress, and injection/gavage-related discomfort, and body weight was recorded during the experiment. Humane endpoints included body-weight loss exceeding 20%, persistent severe dyspnea, inability to access food or water, or severe lethargy. The primary outcome measure was the lung histopathological injury score. No unexpected adverse events were observed during the study.

### 4.4. Histopathological Analysis

Lung and colon tissues fixed in 4% paraformaldehyde were dehydrated, embedded in paraffin, and sectioned at 5 μm. Sections were stained with hematoxylin and eosin (H&E) according to standard procedures. Lung histopathological alterations, including alveolar structural disruption, inflammatory cell infiltration, and alveolar septal thickening, were evaluated under a light microscope. Colon injury was assessed based on epithelial integrity, crypt architecture, mucosal damage, and inflammatory cell infiltration. Histological scores were assigned by investigators blinded to group allocation according to previously described criteria. For histological and immunostaining analyses, slides were coded before image evaluation so that investigators performing scoring and quantification were blinded to treatment group [34,35].

### 4.5. Measurement of Pulmonary Biochemical Indicators

Lung samples were placed in pre-chilled PBS and homogenized on ice, followed by centrifugation at 3500× *g* for 10 min at 4 °C. The resulting supernatants were used to measure the GSH/GSSG ratio, MDA content, SOD activity, total protein concentration, and taurine level. These biochemical parameters were quantified with commercial assay kits according to the corresponding protocols. Values were expressed after correction for total protein content.

### 4.6. Immunohistochemistry

Paraffin-embedded colon sections were deparaffinized, rehydrated through graded alcohols, and subjected to antigen retrieval in 10 mM citrate buffer at pH 6.0. Endogenous peroxidase activity was blocked with 3% H_2_O_2_, and nonspecific binding was reduced by incubation with 5% BSA. The sections were then incubated overnight at 4 °C with primary antibodies against ZO-1 and occludin, followed by HRP-conjugated secondary antibodies. Immunoreactivity was developed using DAB, after which the nuclei were counterstained with hematoxylin. Finally, the slides were dehydrated, coverslipped, and examined under a light microscope. Staining intensity was quantified using ImageJ software, version 1.54p.

### 4.7. Immunofluorescence Staining

Lung sections were deparaffinized, rehydrated, and processed for antigen retrieval using the procedure described above. After incubation with 5% BSA to reduce nonspecific binding, the sections were exposed overnight at 4 °C to primary antibodies targeting SLC6A6 or phosphorylated NF-κB p65. They were subsequently incubated with the appropriate secondary antibodies and counterstained with DAPI to label nuclei. Fluorescence signals were captured using a laser scanning confocal microscope (Carl Zeiss Microscopy GmbH, Jena, Germany). Mean fluorescence intensity was analyzed with ImageJ software, version 1.54p.

### 4.8. Western Blot Analysis

Lung tissues were lysed in ice-cold RIPA buffer containing protease and phosphatase inhibitor cocktails and then centrifuged at 12,000× *g* for 15 min at 4 °C. Protein concentrations of the supernatants were determined by the BCA assay. Equal amounts of protein were separated by SDS-PAGE and electrotransferred onto PVDF membranes. After blocking with rapid blocking buffer at room temperature, membranes were incubated overnight at 4 °C with primary antibodies against SLC6A6, total NF-κB p65, phosphorylated NF-κB p65, IL-1β, IL-6, and β-actin. Detailed antibody information is provided in Supplementary Table S1. Membranes were then incubated with HRP-conjugated secondary antibodies and visualized using enhanced chemiluminescence reagent. Band intensities were quantified using ImageJ 1.54p [36,37].

### 4.9. Quantitative Real-Time PCR

Lung RNA was isolated with a total RNA kit (Xintianweng Biotechnology, Guangzhou, China), and its yield and purity were assessed by NanoDrop. cDNA was generated from 1 μg RNA using a Yeasen reverse transcription kit (Shanghai, China). qPCR was conducted with SYBR Green Master Mix (Yeasen, Shanghai, China) on a real-time PCR platform. TNF-α, IL-1β, and IL-6 transcripts were normalized to β-actin and quantified by the 2^−ΔΔCt^ algorithm. Primers are listed in Supplementary Table S2.

### 4.10. Gut Microbiota Analysis

Gut microbiota analysis was performed in collaboration with Sanshu Company. Colon tissue samples from the Control, CSE, and CSE + FMN 70 groups (n = 3 per group) were collected under sterile conditions and stored at −80 °C. Microbial genomic DNA was extracted using a commercial DNA extraction kit (Omega Bio-tek, Norcross, GA, USA) according to the manufacturer’s instructions. The bacterial 16S rRNA gene V3–V4 region was amplified using universal primers, and purified amplicons were sequenced on an Illumina paired-end platform. Raw reads were quality-filtered, denoised, merged, and taxonomically annotated using the service provider’s standard bioinformatic pipeline. Microbial richness was evaluated using the Chao1 index, and beta diversity was assessed by PCoA. Relative bacterial abundances at the family and genus levels were visualized using stacked bar plots. Differential taxa between the CSE and FMN groups were identified using LEfSe.

### 4.11. Untargeted Metabolomics Analysis

Untargeted metabolomic analysis was conducted in collaboration with Sanshu Company. Fecal samples from the Control, CSE, and CSE + FMN 70 groups (n = 3 per group) were collected at sacrifice and stored at −80 °C. Samples were extracted with prechilled organic solvent, and pooled quality-control samples were prepared to monitor analytical stability. Metabolic profiling was performed using liquid chromatography–tandem mass spectrometry (LC-MS/MS) according to the service provider’s validated protocol. Raw data were processed for peak extraction, alignment, normalization, and metabolite annotation by matching accurate mass and MS/MS spectra against public and in-house databases. PCA, hierarchical clustering, volcano plot analysis, and variable importance in projection analysis were used to characterize metabolic differences. Differential metabolites were further subjected to KEGG pathway enrichment analysis. For metabolite-level comparisons, *p*-values were adjusted using the Benjamini–Hochberg false discovery rate procedure. Adjusted q values were reported together with raw *p*-values.

### 4.12. Correlation Analysis

Spearman’s rank correlation analysis was performed between the relative abundances of selected differential bacterial genera and host indicators using matched individual samples from the Control, CSE, and CSE + FMN 70 groups. The host indicators included the mean densities of ZO-1 and occludin, the GSH/GSSG ratio, MDA level, SOD activity, lung taurine content, normalized SLC6A6 protein levels, the p-NF-κB/NF-κB ratio, and normalized IL-1β and IL-6 protein levels.

### 4.13. Statistical Analysis

Data are presented as mean ± SD. Statistical analyses were performed using GraphPad Prism 10.1.2. Normality of data distribution was assessed via the Shapiro–Wilk test, and homogeneity of variances was verified by Levene’s test. For comparisons among multiple groups, one-way analysis of variance (ANOVA) followed by Tukey’s post hoc test was used when data were normally distributed and variances were homogeneous. When data failed to meet the assumptions of normality or equal variance, the Kruskal–Wallis nonparametric test followed by Dunn’s post hoc test was applied. A value *p* < 0.05 was considered statistically significant. Effect sizes with confidence intervals were not calculated because the study was designed as an exploratory preclinical experiment rather than a confirmatory effect-size estimation study.

## 5. Conclusions

In conclusion, FMN alleviates CSE-induced lung injury in mice. The observed improvement was accompanied by restoration of pulmonary redox–inflammatory homeostasis, preservation of intestinal barrier integrity, remodeling of gut microbiota composition, regulation of taurine metabolism and SLC6A6 expression, and suppression of NF-κB-associated inflammatory activation. Thus, the data support an association between FMN treatment and coordinated changes in the gut microbiota, taurine metabolism, SLC6A6 expression, and NF-κB activation, rather than demonstrating that these components constitute a causal mechanism. These observations provide a basis for targeted mechanistic studies and support further evaluation of natural isoflavone-based interventions for cigarette smoke-associated pulmonary injury.

## Data Availability

Data will be made available on request.

## Supplementary Materials

The following supporting information can be downloaded at: https://www.mdpi.com/article/10.3390/molecules31173090/s1, Figure S1. Molecular structure of formononetin. Table S1. Primary antibodies used for Western blot. Table S2. Primer sequences used for qRT-PCR in mouse lung tissue.

## Abbreviations

ANOVA, analysis of variance; BCA, bicinchoninic acid; BSA, bovine serum albumin; CSE, cigarette smoke extract; DAB, 3,3′-diaminobenzidine; DAPI, 4′,6-diamidino-2-phenylindole; Dex, dexamethasone; ELISA, enzyme-linked immunosorbent assay; FMN, formononetin; GSH, reduced glutathione; GSSG, oxidized glutathione; H&E, hematoxylin and eosin; HRP, horseradish peroxidase; IL-1β, interleukin-1β; IL-6, interleukin-6; KEGG, Kyoto Encyclopedia of Genes and Genomes; LC-MS/MS, liquid chromatography–tandem mass spectrometry; LDA, linear discriminant analysis; LEfSe, linear discriminant analysis effect size; MDA, malondialdehyde; NF-κB, nuclear factor-κB; PBS, phosphate-buffered saline; PCA, principal component analysis; PCoA, principal coordinate analysis; PVDF, polyvinylidene fluoride; qPCR, quantitative real-time polymerase chain reaction; RIPA, radioimmunoprecipitation assay; SD, standard deviation; SDS-PAGE, sodium dodecyl sulfate–polyacrylamide gel electrophoresis; SLC6A6, solute carrier family 6 member 6; SOD, superoxide dismutase; TauT, taurine transporter; TNF-α, tumor necrosis factor-α; VIP, variable importance in projection; ZO-1, zonula occludens-1.
