# Supplementary material for: Formononetin Alleviates Cigarette Smoke Extract-Induced Lung Injury in Mice with Gut Microbiota and Taurine/SLC6A6/NF-κB Modulation
Source: Molecules. 2026 Sep 3;31(17):3090. doi: 10.3390/molecules31173090 (PMC13567225; doi:10.3390/molecules31173090)
Supplement: Supplementary file 1 [file molecules-31-03090-s001.zip › molecules-4423457-supplementary.pdf]

Supplementary Material

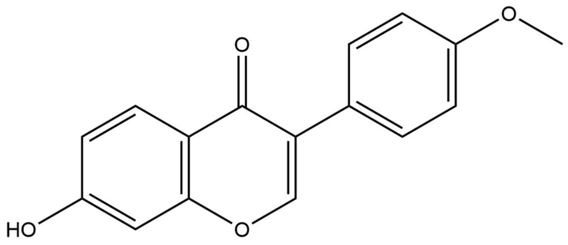

CAS: 485-72-3

Figure S1. Molecular structure of formononetin.

Table S1. Primary antibodies used for Western blot.

Primary antibody	Manufacturer	Molecular weight (kDa)	Dilution
SLC6A6	CUSABIO Biosciences, Wuhan, China	70	1:2000
NF-κB	Nature Biosciences, Hangzhou, China	65	1:1000
p-NF-κB	Nature Biosciences, Hangzhou, China	65	1:1000
IL-1β	Nature Biosciences, Hangzhou, China	17	1:1000
IL-6	Nature Biosciences, Hangzhou, China	24	1:1000
β-Actin	Nature Biosciences, Hangzhou, China	42	1:5000

Table S2. Primer sequences used for qRT-PCR in mouse lung tissue.

Gene	Forward primer	Reverse primer
Tnf	GCGACGTGGAACTGGCAGAAG	GGTACAACCCATCGGCTGGCA
Il6	ATCCAGTTGCCTTCTTGGGACTG	TAAGCCTCCGACTTGTGAAGTGG
Il1b	GAAATGCCACCTTTTGACAGTG	TGGATGCTCTCATCAGGACAG
Actb	TATAAAACCCGGCGGCGCA	TCATCCATGGCGAACTGGTG
